# Effect of biotic and abiotic factors on conidial germination of a saprobic ammonia fungus *Amblyosporium botrytis*

**DOI:** 10.1080/21501203.2017.1363093

**Published:** 2017-08-22

**Authors:** Dinah Corazon M. Licyayo, Akira Suzuki

**Affiliations:** a College of Agriculture and Home Science, Ifugao State University, Lamut, Philippines; b Department of Natural Sciences, Faculty of Knowledge Engineering, Tokyo City University, Tokya, Japan

**Keywords:** Ammonium-nitrogen, pH, temperature, maturation, intercalary type conidia, terminal type conidia

## Abstract

The effects of NH_4_Cl concentration (0.1–1600 mM), temperature (5.0–35.0°C), pH (3.0–11.0), conidial density (2.5 × 10^5^–1.0 × 10^7^ conidia/ml), and conidial maturation (0–21 days) on the conidial germination of the early phase (EP) ammonia fungus *Amblyosporium botrytis* were investigated. The optimum NH_4_Cl concentration for conidial germination was 300 mM. No germination was observed above 1000 mM NH_4_Cl, while low NH_4_Cl concentration (0.1–30 mM) retarded the conidial germination. The conidia did not germinate in distilled water. The effective pH ranges for the conidial germination were from 5.0 to 9.0 with optimum at 8.0. Germination was possible from 5.0°C to 35°C with the optimum at 15°C. These results demonstrate that ammonium-nitrogen concentration and pH are principal factors in the conidial germination of *A. botrytis*. It is expected that the obtained physiological characteristics of *A. botrytis* are suitable to colonize in nitrogen-enriched soil as the EP ammonia fungus.

## Introduction

*Amblyosporium botrytis* is documented on decaying basidiomata, well-rotted wood, plant debris, and dung (Pirozynski 1969) and from the mole midden, known to be an excreting site of *Euroscaptor, Mogera*, or *Urotrichus* (Sagara 1995). *A. botrytis* occurs in forests and weed community enriched with various kinds of nitrogenous materials such as urea, aqua ammonia, uric acid, casein, peptone, sodium glutamate, l-arginine, and ethylendiamine (Sagara 1975). Based on the chemo-ecological characteristics described above, *A. botrytis* is categorized as one of the fungal species in ammonia fungi (Sagara 1975). *A. botrytis* occurs at the early phase (EP) of fungal succession of ammonia fungi (Yamanaka 2003; Imamura and Yumoto 2004). The occurrence of *A. botrytis* would happen through the effects of multiple biotic and abiotic factors. In spite of many ecological researches in *A. botrytis*, its physiological characteristics have not been examined sufficiently (Suzuki 1989, 2006, 2009a, 2009b). In this study, investigations about the effects of biotic and abiotic factors on conidial germination of *A. botrytis* have been performed in order to understand its colonization strategy in the field.

## Materials and methods

### Sampling and germination tests of conidia

#### Sampling of conidia

Stock culture of *A. botrytis* Fres. M006 was grown on malt yeast agar (MYA) medium to obtain conidia. It consists of 10 g of malt extract (Difco), 2 g of yeast extract (Difco), 15 g of agar (Nacalai) and 1000 ml of distilled water. About 8 ml of MYA was placed in φ18 mm test tubes and autoclaved at 120°C for 15 min. After autoclaving, the stock culture of *A. botrytis* was inoculated onto the agar slant and incubated at 25.0 ± 0.3°C in the dark. After 16 days of cultivation (the mycelia cover the whole agar surface area 2 days after inoculation), conidia were washed off from the inoculated agar slant using sterile distilled water. The conidial suspension (pre-suspension) was pipetted from the slant and was decanted through a sterilized fine mesh to separate hyphae and agar particles. The conidial suspension was centrifuged at 500 rpm/min for 10 min. at 5.0 ± .05°C 3 times.

#### Germination tests for conidia

The conidia were rinsed with sterile distilled water under sterile condition and re-suspended in a double strength designated concentration of NH_4_Cl aqueous solution which was filter sterilized using a 0.22-µm pore size cellulose nitrate membrane filter (Advantec) after pH adjustment by NaOH. For control, the sterilized conidia were re-suspended in distilled water. The same procedure was done on the subsequent experiments unless otherwise specified. The conidia were suspended at 3.25 × 10^6^ to 4.7 × 10^6^ conidia/ml in 300 mM NH_4_Cl aqueous solution adjusted at pH 8.0 by NaOH. Twenty milliliter of the conidial suspensions were poured into sterilized 50 ml screwed vial bottle and incubated at 15.0 ± 0.3°C in the dark for 120 h. These conditions were constant on all experiments unless otherwise mentioned.

To determine the suitable maturation period for conidial germination, conidia were gathered from 0, 3, 9, 12, 15, 18, and 21 days after the mycelia covered the whole surface (2 days after inoculation of the MYA slant). To examine the effects of inoculum density on conidial germination, the conidial suspensions were adjusted at 1.0 × 10^7^, 7.7 × 10^6^, 5.5 × 10^6^, 3.2 × 10^6^, 2.0 × 10^6^, 1.2 × 10^6^, 7.1 × 10^5^, and 2.5 × 10^5^ conidia/ml, respectively. Ammonium-nitrogen concentration was examined by suspending the conidia in 0.01, 0.03, 0.1, 0.3, 1, 3, 30, 100, 300, 600, 1000, 1100, 1300, and 1600 mM NH_4_Cl aqueous solution. For control, the sterilized conidia prepared by the procedures described above were re-suspended in distilled water. For the effect of pH on conidial germination, the conidial suspensions were examined separately in 300 mM NH_4_Cl aqueous solution adjusted at various pH, 3.0, 4.0, 5.0, 6.0, 6.5, 7.0, 7.5, 8.0, 8.5, 9.0, 10.0, and 11.0. The aqueous solution adjusted at pH 3.0–5.0 with H_2_SO_4_ or HCl and at pH above 6.0 with NaOH, KOH, or NH_4_OH. The initial and final pHs of the suspensions were measured with a glass electrode. The precision of pH was 0.1. For the examination of temperature, the conidial suspensions were incubated at temperatures from 5.0°C to 35.0°C at 5.0°C interval. The precision of each designated temperature was ±.03°C. In each time of all experiments, one milliliter sample was collected from the incubated conidial suspension at designated time intervals.

### Microscopic observation

**Figure 1.**
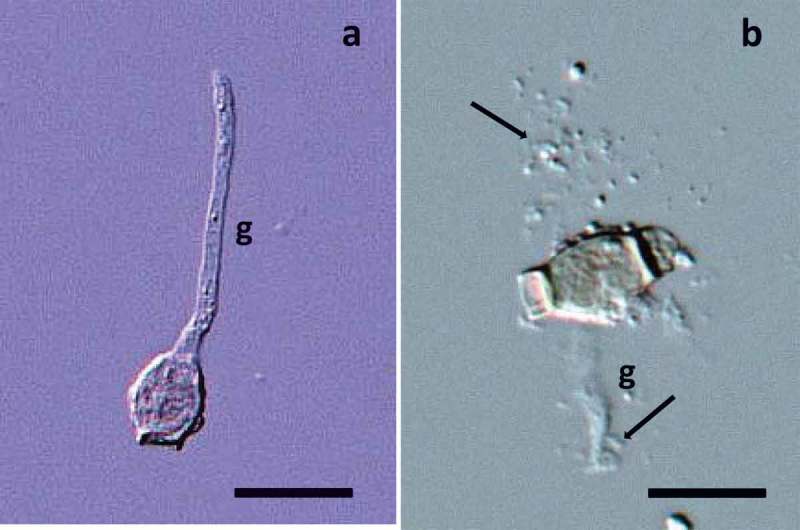
Conidial germination of *A. botrytis*. (a) A germ tube (g) appeared apically from a terminal type conidium after 72 h of the incubation at 15°C in 300 mM NH_4_Cl aqueous solution adjusted at pH 8.0 by NaOH, Bar: 15 μm; (b) an abnormal germination tube appeared laterally from a conidium by 72 h of incubation in 600 mM NH_4_Cl aqueous solution adjusted at pH 8.0 by NaOH. Tiny matters (indicted by arrows) brought out from a germ tube and a conidium itself, Bar: 15 μm.

The 10 µl of conidial suspension smeared from each treatment was mounted on a slide glass. Phloxine B was used to stain the samples. Conidial germination was observed through a light microscope at 400 magnifications, and the conidial density was determined by a hematocytometer. In each treatment, at least more than 50 conidia were examined in random microscopic fields. Conidial germination in the present study is defined as the protrusion of hypha(e) [germtube(s)] discernible under a microscope (Figure 1(a)). Results are shown as the average of seven replicates. The percentage germinations were computed using formula: Conidial germination (%) = number of germinated conidia/ total number of conidia × 100.

### Statistical analysis

Data were analysed by one-way analysis of variance (ANOVA), and significant differences among treatments were determined if necessary, by Tukey–Kramer test at 5% (Statcel 3, The Publisher OMS Ltd, Tokorozawa).

## Results

### Types of conidia

Two types of *A. botrytis* conidia namely intercalary and terminal (Pirozynski 1969) were observed. Intercalary type conidia were formed 5.2–8.5 times of frequency than terminal type ones (Table 1). The germ tube appeared from the all parts of conidia in both types of conidia (cf., Figure 1(a)). The germination percentage of intercalary type conidia showed higher than that of terminal type of conidia (*P *< 0.01) (Table 1). In the following experiments, the percentage germination of conidia was obtained from the samples collected at random from the mixtures of both types of conidia.

**Table 1.** Percentage germination of terminal and intercalary type *A. botrytis* conidia incubated at 15°C for 5 days in 300 mM NH_4_Cl aqueous solution adjusted by different reagents at pH 8.0 by NaOH.

	Percentage germination in each type of conidium		
Ratio (intercalary conidia/terminal conidia)	Intercalary	Terminal	Percentage germination of a mixture of two types of conidia
6.0 ± 0.4	21.5 ± 0.8	17.3 ± 0.5^a^	20.8 ± 0.7
8.5 ± 0.4	56.6 ± 1.4	41.5 ± 1.7^a^	54.1 ± 1.9
5.2 ± 0.2	58.6 ± 0.5	52.4 ± 1.3^a^	57.5 ± 0.6

^a^Significant difference at *P < *0.01 according to Tukey–Kramer test. Numerical numbers in the table are shown by average values with SE *n* = 7.

### Effect of maturation period on conidial germination

**Figure 2.**
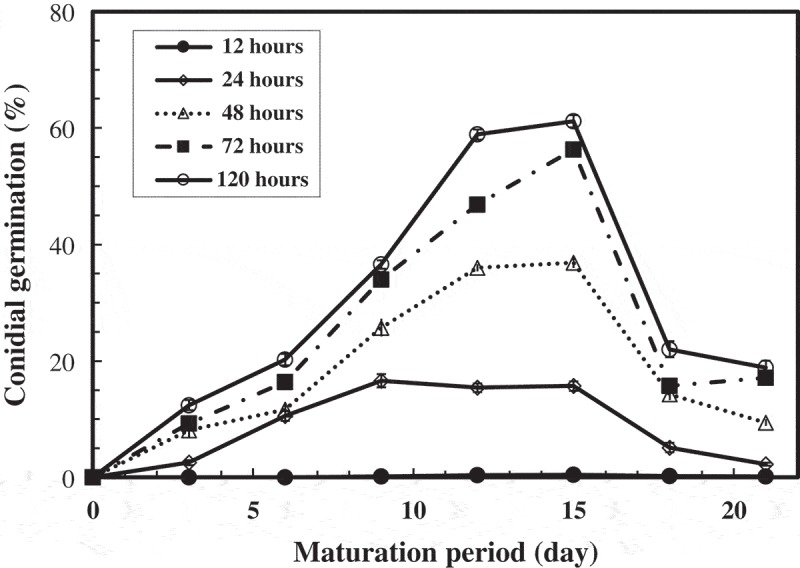
Effect of maturation period on conidial germination. The conidial suspensions obtained from different maturation periods were incubated at 15°C in 300 mM NH_4_Cl aqueous solution adjusted at pH 8.0 by NaOH.

Conidia suspended in 300 mM NH_4_Cl adjusted at pH 8.0 did not germinate after 120 h incubation when they were collected just after the mycelium covered the whole surface of the agar slant (2 days after inoculation). Therefore, two days after the inoculation of culture was shown as 0 day of the maturation period in the following experiments. The conidia gathered after 21 days of the maturation period had 19% germination after 120 h incubation. The maximum conidial germination percentage (59%) was observed from 12 to 15 days of the maturation period (Figure 2). The final pHs of the incubation of the conidial suspensions were 7.7 and 7.8, respectively.

Based on the results, conidia collected from 12 to 15 days of the maturation period were used in all of other experiments.

### Effect of conidial density on conidial germination

**Figure 3.**
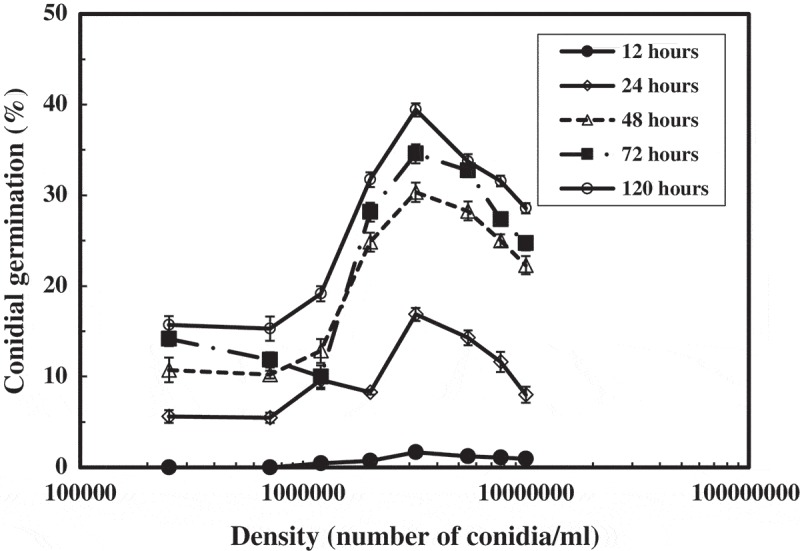
Effect of conidial density on conidial germination. Different densities of the conidial suspensions were incubated at 15°C in 300 mM NH_4_Cl aqueous solution adjusted at pH 8.0 by NaOH.

The conidial density affected the germination ability of the conidia. The highest germination percentage (39.4%) was observed at 3.2 × 10^6^ conidia/ml, while the lowest germination percentage was at 2.5 × 10^6^ and 7.1 × 10^5^ conidia/ml (Figure 3). The germination was firstly observed by the 12 days of cultivation of conidial suspension adjusted higher density than 1.2 × 10^5^ conidia/ml. The conidial germination of the suspensions adjusted less than 7.1 × 10^5^ conidia/ml was observed by 24 h of cultivation. pH of the conidial suspensions were pH 7.8 or 7.9. The decline of pH at different densities of the conidial suspensions was less than 0.3 irrespective of the densities. Based on the results, conidial density was adjusted at 3.2 × 10^6^ conidia/ml in all experiments.

### Effect of NH_4_Cl concentration on conidial germination

Conidial germination was observed from 0.1 mM to 600 mM NH_4_Cl with optimum at 300 mM NH_4_Cl. At 72 h of incubation, percentage germination at 300 mM NH_4_Cl increased to above 60%, whereas that in 0.1–3 mM NH_4_Cl it was only from 0.6% to 5.7% (Table 2). Conidial germination was observed from 12 h of incubation in the suspension treated in 10–100 mM NH_4_Cl and in 0.1–3 mM NH_4_Cl (48 h incubation) and 600 mM NH_4_Cl (72 h incubation) (Table 2). In contrast, conidial germination was not observed in distilled water after 120 h of incubation. Percentage germination of conidia decreased with the decrement of NH_4_Cl concentration ranging from 100 to 0.1 mM (Table 2).

**Table 2.** Effect of various NH_4_-N concentrations on the germination of *A. botrytis* incubated at 15°C.

NH_4_-N	Initial	Percentage germination of conidia at each time (hour)										Final
concentration (mM)	pH	0	6	12	18	24	30	48	72	96	120	pH
1600	8	0 ± *T*	0 ± *T*	0 ± *T*	0 ± *T*	0 ± *T*	0 ± *T*	0 ± *T*	0 ± *T*	0 ± *T*	0±*T*^h^	7.9
1300	8	0 ± *T*	0 ± *T*	0 ± *T*	0 ± *T*	0 ± *T*	0 ± *T*	0 ± *T*	0 ± *T*	0 ± *T*	0±*T*^h^	7.8
1100	8	0 ± *T*	0 ± *T*	0 ± *T*	0 ± *T*	0 ± *T*	0 ± *T*	0 ± *T*	0 ± *T*	0 ± *T*	0±*T*^h^	7.9
1000	8	0 ± *T*	0 ± *T*	0 ± *T*	0 ± *T*	0 ± *T*	0 ± *T*	0 ± *T*	0 ± *T*	0 ± *T*	0±*T*^h^	7.8
600	8	0 ± *T*	0 ± *T*	0 ± *T*	0 ± *T*	0 ± *T*	0 ± *T*	0 ± *T*	3.0 ± 0.6	6.3 ± 1.0	4.7 ± 0.5^e,f^	7.7
300	8	0 ± *T*	0 ± *T*	0.39 ± *T*	2.0 ± 0.2	15.4 ± 0.7	37.1 ± 0.5	58.1 ± 0.9	61.7 ± 1.0	67.2 ± 0.7	67.9 ± 1.0^a^	7.2
100	8	0 ± *T*	0 ± *T*	0.14 ± *T*	1.2 ± 0.2	6.7 ± 0.3	22.1 ± 0.9	22.9 ± 0.6	28.3 ± 1.0	30.3 ± 1.1	31.7 ± 1.0^b^	7.6
30	8	0 ± *T*	0 ± *T*	0.28 ± *T*	0.1 ± 0.9	4.9 ± 0.5	12.0 ± 0.7	17.1 ± 0.7	22.3 ± 1.5	24.0 ± 1.3	26.7 ± 0.9^c^	7.8
10	8	0 ± *T*	0 ± *T*	0.19 ± *T*	0.6 ± 0.2	1.1 ± 0.1	3.6 ± 0.3	8.9 ± 0.5	14.4 ± 1.1	15.1 ± 0.7	16.3 ± 0.5^d^	7.8
0.3	8	0 ± *T*	0 ± *T*	0 ± *T*	0 ± *T*	0 ± *T*	0 ± *T*	3.4 ± 0.6	4.9 ± 0.9	5.1 ± 0.7	5.7 ± 0.7^e^	7.9
0.1	8	0 ± *T*	0 ± *T*	0 ± *T*	0 ± *T*	0 ± *T*	0 ± *T*	1.3 ± 0.3	1.6 ± 0.2	2.0 ± 0.2	2.3 ± 0.2^f,g^	7.9
0.03	8	0 ± *T*	0 ± *T*	0 ± *T*	0 ± *T*	0 ± *T*	0 ± *T*	0.7 ± 0.2	1.0 ± 0.2	1.3 ± 0.2	1.4 ± 0.2g	7.8
0.01	8	0 ± *T*	0 ± *T*	0 ± *T*	0 ± *T*	0 ± *T*	0 ± *T*	0.3 ± 0.1	0.4 ± 0.1	0.5 ± *T*	0.6 ± 0.1^g^	7.8
Control	8	0 ± *T*	0 ± *T*	0 ± *T*	0 ± *T*	0 ± *T*	0 ± *T*	0 ± *T*	0 ± *T*	0 ± *T*	0 ± 0*T*	-

Control: distilled water. *T*: value less than 0.04. Numerical numbers in the table are shown by average value with SE *n* = 7. Columns with the same letter for each treatment are not significantly different at *P < *0.05 according to Tukey–Kramer test.

At 600 mM NH_4_Cl, germination was observed after 72 h incubation. However, conidial abnormality at 32 h incubation was observed (Table 2). The abnormal conidia seemed to have shrunk having lost the normal shape and signs of seemingly ruptured and/or shrunk conidia due to high concentration of ammonium-nitrogen, producing small particles which most likely came from the conidia (Figure 1(b)). The conidial abnormality was not observed at less than 300 mM NH_4_Cl.

The pH of the conidial suspension declined (0.1–0.8) according to the increment of percentage germination. The declining pH (0.1–0.2) was also observed at 120 h of incubation of conidial suspension in 1000–1600 mM NH_4_Cl where the germination was not observed. The lowest final pH (7.2) was observed by 120 h of incubation in 300 mM NH_4_Cl (Table 2).

Based on the results, 300 mM NH_4_Cl was used in all of other experiments.

### Effect of pH on conidial germination

The effective pH ranges for germination of *A. botrytis* were 4.0–9.0, irrespective of pH adjustment reagents, when they were cultured in 300 mM NH_4_Cl. pH optima for the conidial germination was 8.0, irrespective of pH adjustment reagents, and the highest germination percentage (64.3%) was obtained when 300 mM NH_4_Cl was adjusted by KOH. At each pH, the higher germination percentage was obtained when KOH was used for pH-adjustment reagent. The germination percentage showed lower value at each pH adjusted by NaOH and NH_4_OH in that order (Table 3). In the media adjusted by NaOH or KOH at pH 7.5–8.5, germination started after 12 h of incubation, irrespective of pH adjustment reagents. At pH 5.0, only 0.7% of conidia was germinated 120 h after incubation adjusted with HCl or H_2_SO_4_, respectively. At pH 9.0, 2.6%, 3.7%, and 3.7% conidia germinated 120 h after incubation adjusted with KOH, NaOH, and NH_4_OH, respectively (Table 3). The pH declining of the conidia suspensions adjusted at pH 7.5–8.5 was less than 0.6. No declining changes in the pH of the conidial suspensions were observed in NH_4_Cl aqueous solutions at lower and higher pH where conidial germination was not observed (Table 3). Based on the results, all conidial suspensions were adjusted at pH 8.0 by NaOH in all of other experiments.

**Table 3.** Effect of pH on conidial germination of *A. botrytis* incubated at 15°C in 300 mM NH_4_Cl aqueous solution.

pH adjustment	Initial	Percentage germination of conidia at different incubation time (hour)											Final
Reagent	pH	0	6	12	18	24	30	36	48	72	96	120	pH
HCl	3.0	0 ± *T*	0 ± *T*	0 ± *T*	0 ± *T*	0 ± *T*	0 ± *T*	0 ± *T*	0 ± *T*	0 ± *T*	0 ± *T*	0 ± *T*^b^	2.9
	4.0	0 ± *T*	0 ± *T*	0 ± *T*	0 ± *T*	0 ± *T*	0 ± *T*	0 ± *T*	0 ± *T*	0 ± *T*	0 ± *T*	0 ± *T*^b^	5.0
	5.0	0 ± *T*	0 ± *T*	0 ± *T*	0 ± *T*	0 ± *T*	0 ± *T*	0 ± *T*	0 ± *T*	0 ± *T*	0 ± *T*	0.7 ± 0.2^a^	4.8
H_2_SO_4_	3.0	0 ± *T*	0 ± *T*	0 ± *T*	0 ± *T*	0 ± *T*	0 ± *T*	0 ± *T*	0 ± *T*	0 ± *T*	0 ± *T*	0 ± *T*^b^	3.0
	4.0	0 ± *T*	0 ± *T*	0 ± *T*	0 ± *T*	0 ± *T*	0 ± *T*	0 ± *T*	0 ± *T*	0 ± T	0 ± *T*	0 ± *T*^b^	4.0
	5.0	0 ± *T*	0 ± *T*	0 ± *T*	0 ± *T*	0 ± *T*	0 ± *T*	0 ± *T*	0 ± *T*	0 ± *T*	0 ± *T*	0.7 ± 0.2^a^	5.0
NH_4_OH	6.0	0 ± *T*	0 ± *T*	0 ± *T*	0 ± *T*	0 ± *T*	0 ± *T*	0 ± *T*	0 ± *T*	0.3 ± 0.2	0.4 ± 0.2	1.1 ± 0.3^c^	5.7
	6.5	0 ± *T*	0 ± *T*	0 ± *T*	0 ± *T*	0 ± *T*	0 ± *T*	0 ± *T*	0 ± *T*	0.6 ± 0.2	1.0 ± 0.2	1.1 ± 0.3^c^	6.4
	7.0	0 ± *T*	0 ± *T*	0 ± *T*	0.4 ± 0.2	0.9 ± 0.3	1.4 ± 0.2	2.9 ± 0.3	3.0 ± 0.2	5.7 ± 0.3	9.6 ± 0.7	16.1 ± 0.6^b^	6.6
	7.5	0 ± *T*	0 ± *T*	0.1 ± 0.1	0.6 ± 0.2	1.1 ± 0.3	1.3 ± 0.2	2.6 ± 0.3	3.1 ± 0.3	7.3 ± 0.4	9.7 ± 0.7	15.4 ± 0.9^b^	7.4
	8.0	0 ± *T*	0 ± *T*	0.7 ± 0.2	1.9 ± 0.3	4.4 ± 0.2	10.3 ± 0.6	11.9 ± 0.7	15.1 ± 0.7	19.4 ± 0.5	19.1 ± 0.8	20.6 ± 0.8^a^	7.7
	8.5	0 ± *T*	0 ± *T*	0.7 ± *T*	0.1 ± 0.1	0.2 ± 0.1	1.1 ± 0.3	5.7 ± 1.0	9.7 ± 0.5	14.0 ± 1.3	16.0 ± 1.2	17.0 ± 0.6^b^	8.2
	9.0	0 ± T	0 ± T	0 ± T	0 ± T	0 ± T	0 ± T	0 ± T	0.1 ± 0.5	0.7 ± 0.3	1.0 ± 0.2	2.6 ± 0.4 c	8.7
	10.0	0 ± *T*	0 ± *T*	0 ± T	0 ± T	0 ± T	0 ± T	0 ± T	0 ± T	0 ± T	0 ± T	0 ± *T*^d^	10.0
	11.0	0 ± *T*	0 ± *T*	0 ± *T*	0 ± *T*	0 ± *T*	0 ± *T*	0 ± *T*	0 ± *T*	0 ± *T*	0 ± *T*	0 ± *T*^d^	11.0
NaOH	6.0	0 ± *T*	0 ± *T*	0 ± *T*	0 ± *T*	0 ± *T*	0 ± *T*	0 ± *T*	0 ± *T*	0 ± *T*	0 ± *T*	0.7 ± 0.2^e^	5.8
	6.5	0 ± *T*	0 ± *T*	0 ± *T*	0 ± *T*	0 ± *T*	0 ± *T*	0 ± *T*	0 ± *T*	0 ± *T*	0.3 ± 0.2	0.6 ± 0.2^e^	6.3
	7.0	0 ± *T*	0 ± *T*	0 ± *T*	0 ± *T*	0 ± *T*	1.9 ± 0.3	2.4 ± 0.4	3.3 ± 0.4	4.3 ± 0.3	6.4 ± 0.6	11.3 ± 0.6^d^	6.5
	7.5	0 ± *T*	0 ± *T*	0.1 ± 0.1	1.0 ± 0.2	10.0 ± 0.9	14.3 ± 0.7	14.6 ± 0.7	34.0 ± 1.4	33.6 ± 1.5	37.0 ± 1.0	43.3 ± 1.0^b^	7.2
	8.0	0 ± *T*	0 ± *T*	0.9 ± 0.3	3.9 ± 0.3	18.0 ± 0.8	28.3 ± 0.1	28.9 ± 0.9	36.3 ± 0.6	40.7 ± 0.7	46.1 ± 0.7	51.0 ± 1.0^a^	7.4
	8.5	0 ± *T*	0 ± *T*	0.3 ± 0.2	1.4 ± 0.2	8.0 ± 0.4	9.7 ± 0.5	13.7 ± 0.7	22.9 ± 1.1	25.1 ± 0.6	28.0 ± 1.1	30.7 ± 1.3^b^	8.1
	9.0	0 ± *T*	0 ± *T*	0 ± *T*	0 ± *T*	0 ± *T*	0 ± *T*	0 ± *T*	0.1 ± *T*	0.1 ± 0.1	1.0 ± 0.2	3.7 ± 0.5^e^	8.6
	10.0	0 ± *T*	0 ± *T*	0 ± *T*	0 ± *T*	0 ± *T*	0 ± *T*	0 ± *T*	0 ± *T*	0 ± *T*	0 ± T	0 ± T^e^	10.0
	11.0	0 ± T	0 ± T	0 ± T	0 ± T	0 ± T	0 ± T	0 ± T	0 ± T	0 ± T	0 ± T	0 ± *T*^e^	11.0
KOH	6.0	0 ± *T*	0 ± *T*	0 ± *T*	0 ± *T*	0 ± *T*	0 ± *T*	0 ± *T*	0 ± *T*	0 ± *T*	3.6 ± 0.2	4.1 ± 0.5 ^f^	5.8
	6.5	0 ± *T*	0 ± *T*	*0 ± T*	*0 ± T*	0 ± *T*	0 ± *T*	1.0 ± 0.2	9.6 ± 0.5	15.4 ± 0.3	15.7 ± 0.7	17.1 ± *T*^e^	6.1
	7.0	0 ± *T*	0 ± *T*	0 ± *T*	0 ± *T*	0 ± *T*	0.6 ± 0.2	1.0 ± 0.2	12.1 ± 0.3	20.9 ± 1.1	24.9 ± 1.1	29.4 ± 0.8^d^	6.6
	7.5	0 ± *T*	0 ± *T*	0.2 ± 0.2	0.9 ± 0.1	6.6 ± 1.0	7.7 ± 0.7	9.4 ± 0.8	26.9 ± 0.7	29.7 ± 0.8	49.7 ± 1.1	51.7 ± 0.9^c^	7.3
	8.0	0 ± *T*	0 ± *T*	0.7 ± 0.2	1.3 ± 0.2	9.4 ± 1.0	13.4 ± 0.9	13.6 ± 0.8	31.1 ± 0.9	35.7 ± 0.3	55.1 ± 0.7	64.3 ± 0.7^a^	7.6
	8.5	0 ± *T*	0 ± *T*	0.1 ± 0.1	1.7 ± 0.2	9.1 ± 0.6	12.3 ± 0.6	15.4 ± 0.9	28.6 ± 1.1	29.7 ± 1.2	29.7 ± 1.0	39.1 ± 0.6^b^	8.1
	9.0	0 ± T	0 ± T	0 ± *T*	0 ± *T*	0 ± *T*	0 ± t	0 ± *T*	0.1 ± *T*	0.3 ± 2.3	1.0 ± *T*	3.7 ± 0.5 f	8.7
	10.0	0 ± *T*	0 ± *T*	0 ± *T*	0 ± *T*	0 ± *T*	0 ± *T*	0 ± *T*	0 ± *T*	0 ± *T*	0 ± *T*	0 ± *T*^g^	10.0
	11.0	0 ± *T*	0 ± *T*	0 ± *T*	0 ± *T*	0 ± *T*	0 ± *T*	0 ± *T*	0 ± *T*	0 ± *T*	0 ± *T*	0 ± *T^g^*	11.0

*T*: less than 0.04. Average value with SE. Numerical numbers in the table are shown by average value with SE, *n* = 7. Columns with the same letter for each treatment are not significantly different at *P < *0.05 according to Tukey–Kramer test.

### Effect of temperature on conidial germination

**Figure 4.**
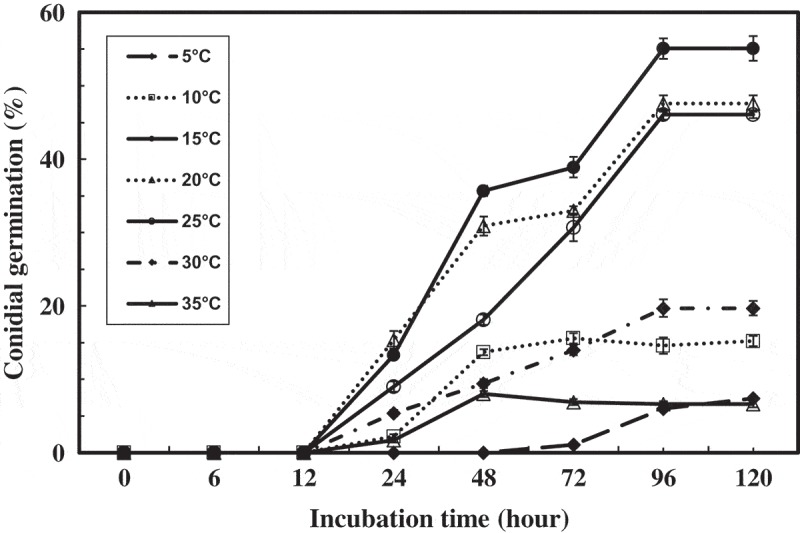
Effect of temperature on conidial germination. The conidial suspensions were incubated at different temperatures in 300 mM NH_4_Cl aqueous solution adjusted at pH 8.0 by NaOH.

Conidial germination was observed at all temperatures when conidia were incubated in 300 mM NH_4_Cl at pH 8.0. The germination started after 12 h of incubation or a little bit earlier from 15°C to 35°C. The germination started 24 h of incubation and 48 h of incubation at 10°C. The maximum percentage germination (55.1%) was observed at 15°C. Conidial germination at a low temperature and at a high temperature resulted in low percentage germination. At 5°C, only 0.4% germination was observed after 48 h and a slow germination progress of 7.4% after 120 h (Figure 4). pH declining by the incubation at different temperatures was less than 0.3, irrespective of the temperature.

Based on the results, 15°C was used in all of other experiments.

## Discussion

Conidia collected earlier (0–9 days) and later (18–21 days) showed poor germination ability when grown on 300 mM NH_4_Cl (Figure 2). It suggests that conidia keep a certain germination ability irrespective of the maturation periods.

In *A. botrytis*, germination percentage of conidia showed maximum value at 3.2 × 10^6^ conidia/ml and gradually decreased with the decrement and increment of the density (Figure 3). This means that discharge of conidia clustered at a suitable degree could be advantage for the colonization of *A. botrytis* in the field.

The effective concentration of NH_4_Cl for the conidial germination in the EP of a saprobic ammonia fungus *A. botrytis* was 0.1–600 mM with optimum at 300 mM (Table 2). Conidia of *A. botrytis* did not germinate above 1000 mM NH_4_Cl (Table 2) and in distilled water. It means that ammonium-nitrogen is a principal environmental factor for the conidial germination, and about 600 mM NH_4_^+^ is inhibitive for conidial germination of *A. botrytis* because conidium abnomality was observed when it was incubated at 600 mM NH_4_Cl (Figure 1(b)).

The optimum and upper limit of NH_4_Cl concentration for vegetative growth of *A. botrytis* is 300–1100 mM (Licyayo and Suzuki 2006). This indicates that the effective range of ammonium-nitrogen concentration for conidial germination was narrower than that for vegetative growth The effective pH ranges for conidial germination and that for vegetative growth are 5.0–9.0 with optimum at 8.0 (Table 3) and 4.0–9.0 with optimum at 7.0–8.0 (Yamanaka 2003), respectively It indicates that the effective pH ranges and pH optimum for conidial germination are a little bit higher than those for vegetative growth. Effective ranges of NH_4_Cl concentration and those of pH for basidiospore germination of the late stage of EP fungi *Coprinopsis* spp. are 0.1–1000 mM with optimum 10–100 mM (Raut et al. 2011). Effective ranges of pH for basidiospore germination of the late stage of EP fungi *Coprinopsis phlyctidospora* complex are 6.0–9.5 with optimum at pH 8.0–8.5 (Raut et al. 2011). Effective range of NH_4_Cl concentration for basidiospore germination of an ectomycorrhizal fungus *Hebeloma vinosophyllum* which occurs at the late phase (LP) of the succession are 10–500 mM with optimum 100 mM (Deng and Suzuki 2008). Effective range of pH for basidiospore germination of *H. vinosophyllum* is 4.5–9.0 with optimum 8.0 (Deng and Suzuki 2008). The pH optima for basidiospore germination of *Coprinopsis* spp. in *C. phlyctidospora* complex and *H. vinosophyllum* are similar to that for conidial germination of *A. botrytis*. Ammonium-nitrogen concentration and pH of urea-treated soil are around 10 mg N/g dry soil (roughly equivalent to 170 mM NH_4_-N) and 9, respectively (Suzuki et al. 2002).

These findings show that the obtained physiological characteristics of *A. botrytis* were suitable for its colonization in nitrogen-enriched soil at the EP of the succession, and it can potentially colonize by the conidia formed by the first flush. It is expected that ammonium-nitrogen concentration and pH of the soil would be principal factors for the colonization of *A. botrytis* in the field, but these abiotic factors are not sufficient to explain the successive appearance of reproductive structures (succession).

In the field, *A. botrytis* occurs by urea treatment both in summer and winter (Sagara et al. 2008). The occurrence of *A. botrytis* in all seasons would be partially explained by the ability of conidial germination at the wide range of temperatures.

Conidial suspension of *A. botrytis* in 300 mM NH_4_Cl aqueous solution (pH adjusted at 7.5–8.0) showed higher germination percentages when conidial suspension was adjusted to designated pH by KOH, NaOH, and NH_4_OH, in that order (Table 3). In contrast, germination percentage of basidiospore suspension of *Coprinopsis* p. ssp. in *C. phlyctidospora* complex and *H. vinosophyllum* in NH_4_Cl aqueous solution adjusted at various pH showed similar germination percentages, irrespective of pH adjustment reagents, KOH, NaOH, and NH_4_OH (Deng and Suzuki 2008; Raut et al. 2011). It suggests that K and Na synergistically stimulate conidial germination of *A. botrytis*. There are at least two types of spores in ammonia fungi according to the response to germination, that is, (1) spore germination stimulated only by ammonium-nitrogen as observed in *Coprinopsis* spp., in *C. phlyctidospora* complex and *H. vinosophyllum* and (2) that stimulated by ammonium-nitrogen synergistically with potassium or sodium as observed in *A. botrytis. A. botrytis* started to germinate within 12 h of incubation (Table 2). It indicates the EP fungus *A. botrytis* has the suitable physiological characteristics to colonize in the filed as a S-R strategist according to the definition by Cooke and Rayner (1984).

In conclusion, the essential mechanism to explain the successional occurrence of *A. botrytis* in the field would be its adaptation to high concentration of ammonium-nitrogen associated with a neutral to alkaline condition at conidial germination and vegetative growth by the physiological characteristics observed at conidial germination as well as those observed at vegetative growth.
